# Osteopontin: Its Properties, Recent Studies, and Potential Applications

**DOI:** 10.3390/ijms26125868

**Published:** 2025-06-19

**Authors:** Büşra Karasalih, Hatice Duman, Mikhael Bechelany, Sercan Karav

**Affiliations:** 1 Department of Molecular Biology and Genetics, Çanakkale Onsekiz Mart University, Çanakkale 17100, Türkiye; busrakarasalih@gmail.com (B.K.); hatice.duman@comu.edu.tr (H.D.); 2 Institut Européen des Membranes (IEM), UMR 5635, University Montpellier, ENSCM, CNRS, F-34095 Montpellier, France; 3 Functional Materials Group, Gulf University for Science and Technology (GUST), Masjid Al Aqsa Street, Mubarak Al-Abdullah 32093, Kuwait

**Keywords:** osteopontin, biomarker, cancer, infant health, intestinal development, temporal expression of cytokines, immunomodulator, milk

## Abstract

OPN is a phosphorylated glycoprotein found in all vertebrate organisms and expressed in many tissues and secretions. It is a pleiotropic protein that plays diverse roles in various pathological and physiological processes. OPN is involved in many tissue transformation events such as intestinal and brain development, the regulation of immune system activity, immune cell activation, and inflammatory responses. This protein increases the functionality of the digestive system by regulating the intestinal microbiome and may help strengthen the intestinal barrier. OPN can also influence cognitive development and behavior. In addition, its recent association with cancer has gained critical importance. The increased expression of OPN has been observed in many cancer types, which may promote tumor cell metastasis. OPN is also effective in bacterial interaction and infections; it can prevent bacterial adhesion, supporting the development of new therapeutic approaches for oral care. Furthermore, the supplementation of OPN in infant formula has positively influenced the immune and intestinal health of infants. Many recent studies have focused on these aspects. This article provides a review and comparison of the existing knowledge on the structure and functions of OPN. It emphasizes how milk-derived OPN impacts human and infant health and disease.

## 1. Introduction

Osteopontin (OPN) is a multifunctional protein present in many human tissues and body fluids including bone, skin, urine, milk, and blood [1]. OPN is generally described as a phosphoprotein associated with cellular transformation, and it was initially identified in the extracellular matrix of bovine bone [2,3]. Oldberg et al. introduced the term OPN (“osteo” from Greek meaning bone, and “pontin” from Latin “pons”, meaning bridge) due to its capability to form a bridge between the cells and mineral components of bone [4]. OPN is found in small integrin-binding ligand *N*-linked glycoprotein (SIBLING) [5]. OPN is composed of 314 amino acids that are abundant in aspartate, glutamate, and serine residues [6], with a molecular weight range of 44–75 kDa [3] and is encoded by the human SPP1 gene. The OPN-encoding gene is located on chromosome 4 region 22 and consists of seven exons. OPN contains several preserved structural domains: the RGD domain (arginine-glycine-aspartate), the SVVYGLR domain (serine-valine-valine-tyrosine-glutamate-leucine-arginine), the heparin-binding domain, the calcium-binding domain, cleavage sites for matrix metalloproteinases, and the CD44 receptor-binding domain [5]. OPN, which attaches to integrin and CD44 receptors, participates in cellular signaling and cell–matrix interactions [7].

When OPN, bound to more than one integrin receptor, is stimulated by these receptors, it increases the migration and invasion of cells [8]. OPN is expressed in many species, including humans and rodents [9,10,11,12,13]. OPN-expressing cells include osteoclasts; osteoblast; breast, kidney, and skin epithelial cells; nerve cells; vascular smooth muscle cells; and endothelial cells [9,14,15,16,17]. Active immune system cells, such as T cells, natural killer cells (NK cells), macrophages, and Kupffer cells, produce OPN, which is then dispersed into body fluids [18,19,20].

In physiological conditions, the amount of OPN expression is lower, and it plays crucial roles in regulating biomineralization, wound healing, and developmental processes. In pathological settings, OPN frequently shows considerable increases and exhibits broad-spectrum actions in various inflammatory, autoimmune, degenerative, fibrotic, and oncologic disorders, including cancer, diabetes, stroke, kidney damage, and cardiac fibrosis [21]. Additionally, OPN may exert beneficial effects across various biological contexts, such as the immune system [22,23,24,25], tissue repair, bone [26,27] and dental health [28,29,30], and neurological conditions [31,32,33]. Furthermore, it has also been proposed as a biomarker in a range of mammalian diseases [34,35,36,37,38,39,40,41].

This review aims to provide general information about OPN and demonstrate its potential in various areas. It begins with an overview of its structure and chemical properties, followed by the biosynthesis pathway of OPN. Subsequently, the effects of environmental and genetic factors on OPN expression levels are explained and discussed; then, the diverse biological functions of OPN are highlighted in both physiological and pathological contexts. Finally, particular attention is given to the nutritional potential of OPN, including its implications in health and disease. Through an integrated analysis of current findings, this article provides a broad and in-depth understanding of OPN’s multifaceted roles and potential in biology and health sciences.

## 2. Structure and Chemical Properties of Osteopontin

This section outlines the structural and chemical characteristics of OPN, focusing on its amino acid composition; post-translational modifications, particularly phosphorylation; and its functional domain interactions. It also highlights interspecies biochemical differences and OPN’s role in cell signaling and mineralization.

**Figure 1:**
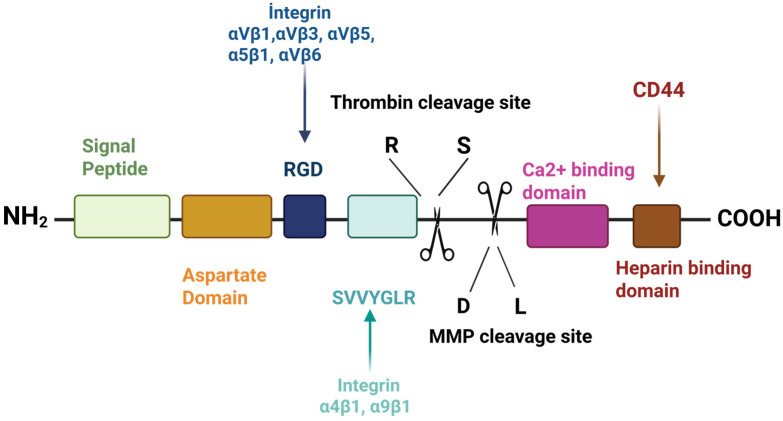
Structure of osteopontin [42,54].

OPN comprises approximately 314 amino acids and includes both O-linked and N-linked oligosaccharides containing a significant amount of aspartic acid, glutamic acid, and serine, along with about 30 monosaccharides (Figure 1). The molecular weight of OPN varies between 44 and 75 kDa [42,43], primarily due to various post-translational modifications (PTMs), such as glycosylation, phosphorylation, and sulfation, which significantly enhance its structural and functional diversity [44,45]. PTMs, especially phosphorylation, critically affect OPN’s function and represent key mechanisms that regulate cellular processes associated with OPN, including interactions with various receptors and physiological processes like mineralization [46]. For instance, phosphorylated OPN facilitates osteoclasts’ adhesion to the surface via the RGD motif and promotes bone resorption, whereas these functions are diminished in dephosphorylated forms [47,48]. In addition, the immune-related roles of OPN, such as macrophage activation, interleukin-12 production, and trophoblastic cell migration, are also dependent on phosphorylation [49,50]. The degree of phosphorylation is crucial in determining whether OPN promotes or suppresses hydroxyapatite crystals’ formation [51,52]. Given that phosphorylation sites are determined by the amino acid sequence, species-specific variations in the OPN structure may influence its functional properties, including its phosphorylation potential [53].

Studies indicate differences in amino acid composition between mature human and bovine OPN. Mature human milk OPN (hmOPN) contains 298 amino acids, while bovine milk OPN (bmOPN) includes 262 amino acids. This difference arises from the absence of 22 residues in bovine OPN, corresponding to amino acids 188–209 in mature human OPN. The sequences of bovine and mature human OPN are similar in 182 locations. Both types of OPN exhibit high contents of aspartic acid, glutamic acid, and phosphorylated residues, contributing to their acidic character. The isoelectric point (pI) of OPN, measured without phosphorylation and found to be 4.1, further confirms its acidic nature [55]. In addition to these compositional characteristics, OPN contains several functionally important domains that contribute to its diverse biological roles, including an RGD-binding area, two additional integrin-binding areas, one thrombin-binding area, and a calcium-binding area. It is cleaved by matrix metalloproteinases 3 and 7 (MMP-3,7), generating functionally distinct N-terminal and C-terminal fragments. The N-terminal contains integrin receptors, while the C-terminal includes two heparin-binding areas and interacts with CD44 receptors [56]. The RGD and SVVYGLR sequences, which are two integrin-binding areas, specifically interact with integrins upon proteolysis. Additionally, bovine OPN also contains the SVAYGLK sequence [44,57].

BmOPN contains around 25 phosphorylated amino acids, primarily made up of 34 phosphoserines and 2 phosphothreonines, located at 28 distinct phosphorylation regions. One study identified OPN as the most phosphorylated protein in the human colostrum [58]. Protein phosphorylation, which is a PTM, is critical for regulating cellular functions, activating signal transmission pathways, and is particularly important in the context of OPN; therefore, identifying the kinases responsible for this modification is essential for understanding its biological roles. During this process, the kinases responsible for the phosphorylating of certain proteins were not identified. Subsequently, FAM20C was found in Golgi lumen and classified as a mammary gland casein kinase due to its ability to phosphorylate all milk proteins. Mutations in the kinase can lead to structural abnormalities in bone [59]. FAM20C is the main kinase responsible for phosphorylation; it acts in mineralized tissues. It phosphorylates OPN on serine and threonine residues, especially along acidic motifs, thus directly affecting the biological activity of OPN. For example, a mutation that can occur in this kinase can disrupt the phosphorylation of OPN, leading to a disease such as Raine syndrome, which is accompanied by disorders of bone mineralization. As a result, FAM20C has an important role in regulating the PTMs of OPN [59,60].

### Biosynthesis of Osteopontin

OPN, a phosphoprotein first identified by Senger in 1979 and named by Franzen in 1985, is encoded by the SPP1 gene located on chromosome 4q22.1. This gene spans approximately 7.7 kb and consists of seven exons [2,5]. The unmodified human OPN protein has a molecular weight of around 35 kDa; however, extensive post-translational modifications (PTMs) increase its apparent weight to 40–80 kDa on SDS-PAGE [42,58].

The structure of OPN is characterized by predominant serine phosphorylation and the presence of acidic amino acids, which gives it a negative charge, and it also contains two accepted heparin-binding sites and a calcium-binding site in OPN [61,62]. Additionally, the specific RGD sequences can be identified, linked, and expressed through integrins located on the cell surface, and these unique structural features are closely related to the biosynthesis process of OPN. The in vitro biosynthesis of OPN begins with the cloning of cDNA that encodes a bone-specific sialoprotein, which is 1473 base pairs long and encodes a protein with 317 amino acid residues. The sialoprotein mRNA includes a coding region with the AUG initiation codon and the signal peptide sequence [4]. The regulation of OPN biosynthesis occurs at the transcriptional level of the SPP1 gene, with alternative splicing contributing to the generation of distinct isoforms, such as in OPNa, OPNb, and OPNc. OPNa contains exons 2–7, while OPNb lacks exon 5, and OPNc lacks exon 4 [63]. Importantly, all human alternative splicing isoforms retain a signal sequence that preserves critical regions of the protein, including the calcium-binding domain and thrombin-binding sites. For instance, amino acid sequences containing SVYGLR and RGD, along with cleavage sites for matrix metalloproteases (MMPs), are included. This represents an alternative translational isoform, which is one of the OPN isoforms. The SPP1 gene blocks the secretion of OPN when the signal sequence is removed or not used to create alternative translational isoforms and allows the production of intracellular OPN (iOPN), which results in secreted OPN (sOPN) if the SPP1 transcript contains the signal sequence [64]. OPN is present in multiple isoforms that arise through alternative translation initiation and alternative splicing mechanisms. It offers structural and functional diversity due to differences in the exon structure. Alternative splicing produces isoforms that can vary according to PTMs, the ability to be cleaved by proteases, and the capacity to interact with different receptors [65]. For example, some OPN isoforms lack certain integrin-binding domains, which affects their function in cell adhesion and signal transduction. Thus, this allows OPN to be involved in various biological processes in a tissue- and context-specific manner [64].

The polypeptide chain formed as a result of translation undergoes PTM. Phosphorylation occurs specifically depending on the characteristics and type of tissue. OPN is exposed to various types, including serine and threonine phosphorylation. For example, hmOPN has undergone a high degree of phosphorylation. In addition, hmOPN contains 36 phosphorylation sites, including 34 phosphoserine, 2 phosphothreonine, 5 O-glycosylated threonine, and N-glycosylation. In total, 29 of the phosphorylation sites are found in the region, also known as mammalian milk casein kinase or Golgi kinase, and 6 phosphorylations are present in the casein kinaseII (CK2) sequence. Also, five threonine residues, which have undergone Thr118, Thr122, Thr127, Thr131, and Thr136, have been shown to have undergone O-glycosylation [58]. Studies conducted on hmOPN show that there are characteristics of O-glycosylation occurring in these five threonine residues. The resulting O-glycosylation has been shown to provide resistance to the pepsin digestion of integrin-binding motifs and to aid in cell adhesion [66]. In another study, it was found that phosphorylation and glycosylation occurring at modification sites located in and near the integrin-binding sites can modulate the interaction of OPN and integrin; for example, the phosphorylation of Ser146 has been found to critically reduce the RGD-mediated αvβ3 integrin interaction [66,67]. These modifications significantly affect the structure and functions of OPN. In particular, phosphorylation is highly effective in regulating cellular processes [45], for example, support bone resorption, by facilitating the adhesion of osteoclasts to the surface. However, dephosphorylated forms cannot carry out these functions [47,48]. Additionally, the immune system functions of OPN such as macrophage activation, interleukin-12 (IL-12) production, and trophoblastic cell migration are also dependent on phosphorylation [49,50]. PTMs are important for the regulation of biological properties such as mineralization an immune response [46].

## 3. Effects of Environmental Conditions and Genetic Factors on OPN Levels

The World Health Organization (WHO), the United Nations Children’s Fund, and the American Academy of Pediatrics support the idea that human milk is the most effective and the only food source that is “worth gold” during the first 6 months of life for newborns [68,69]. Breast milk is a vital source of nutrition and is the main nutrient that babies physiologically need for growth and development [70,71,72,73,74]. Breastfed babies are less likely to develop certain health problems than non-breastfed babies, and breast milk has a protective effect on intestinal health and development [75,76,77]. At the same time, it also has a potential impact on cognitive development [78]. This benefit is attributed to many bioactive elements such as proteins and peptides with antimicrobial and immune-stimulating properties, including lactoferrin, lactoperoxidase, lysozyme, and IgA found in human milk. Among these components, OPN plays a significant role with functions including immune system support, neurodevelopment, and protecting against [24] disease-causing pathogens [79]. Some studies have shown that the OPN concentrations vary across different geographic regions [70,80,81]. At the same time, other studies have found differences in OPN levels even among mothers from the same region [81,82]. Several studies conducted in China have examined the variation in OPN concentrations in breast milk [80,83,84]. In one study, the components of breast milk were analyzed by ELISA and the protein content was tested by the BCA method. A total of 318 milk samples were collected from 106 mothers. These samples were collected from mothers with different breastfeeding periods. OPN levels were measured as 343.2 ± 163.5 mg/L, 228.4 ± 121.5 mg/L, and 204.8 ± 100.6 mg/L in mothers at 1–14 days, 2–4 months, and 5–7 months post-partum, respectively. The results showed that the amounts of OPN levels were highest during the first 2 weeks and decreased over time. In other words, the amount of OPN in colostrum and transition milk was higher than in mature milk. In addition, the study found that the amount of OPN in milk was significantly associated with the mother’s muscle mass as well as with fetal factors during different breastfeeding periods [80]. A study conducted in 2014 similarly examined OPN levels, but in that study, the amounts of OPN in the umbilical cord blood were evaluated. As a result, it was found that the amount of OPN was inversely related to the mother’s gestational age, and this association was statistically significant [85]. In another study conducted in China, milk samples were taken from 105 breastfeeding women after childbirth. Samples were collected over a period of 1–5 days, 8–14 days, 1 month, and 6 months, and the average OPN concentrations were measured as 718 mg/L, 586 mg/L, 450 mg/L, and 236 mg/L, respectively, by using ultra-performance liquid chromatography combined with mass spectrometry (UPLC-MS/MS). The study found that the amount of OPN decreased during breastfeeding, and that milk proteins may change depending on variables related to childbirth [83]. Another study conducted in China examined OPN levels in the milk of mothers who gave birth at term and pre-term. Milk samples were taken from 131 mothers who gave birth at different times. These times were divided into four groups: term, medium-late pre-term (MPT), very pre-term (VPT), and extreme pre-term (EPT). The samples were collected on the 7th, 14th, 28th, and 120th days after birth. OPN levels were determined by UPLC-MS/MS and multiple reaction monitoring (MRM) techniques. Similar to previous studies, this study found that the amount of OPN decreased over time. Pre-pregnancy body mass index (BMI) was found to affect colostrum and transitional milk, suggesting that BMI is one of the maternal factors influencing OPN levels. As a result, it was concluded that the amount of OPN may vary depending on maternal factors, and that differing amounts of OPN may influence infant growth and development, indicating a statistically significant association [84]. Another study was conducted in America. Although human milk contains an abundant amount of OPN, it was not found in infant formula. The study demonstrated that supplementing infant formula with bmOPN leads to improved immune outcomes for infants. The concentration of hmOPN was measured by the ELISA method. Milk samples were collected at 1–7 days, 8–14 days, 1 month, 4 months, and 12 months post-partum. The OPN levels were found to be 178.0 ± 17.9 mg/L, 134.8 ± 18.5 mg/L, 65.8 ± 13.7 mg/L, 55.9 ± 13.8 mg/L, and 48.3 ± 10.2 mg/L, respectively, again showing a statistically significant decrease over time [81]. Another study conducted in America was to investigate milk proteins and the specific glycosylation patterns of these proteins, and to analyze changes during the lactation process. A total of 231 milk samples were taken from 33 mothers across seven different breastfeeding periods and were analyzed by tandem mass spectrometry. After collecting the colostrum samples, the amounts of OPN were recorded at weeks 2, 5, 10, 13, 17, and 24, with average values of 180, 320, 300, 260, 190, 200, and 150, respectively. In this study, the amount of OPN generally decreased over time, although the level at week 2 was approximately twice that of the colostrum milk [82]. In a study conducted in Türkiye, the association between the amount of OPN in breast milk and maternal factors was investigated, as well as the importance of OPN levels for neonatal health. Samples were taken from a total of 85 mothers, who were grouped according to various characteristics. These groups were categorized based on age, birth method, BMI, weight gain during pregnancy, and smoking status. Firstly, four groups were formed based on ages. The OPN levels in these groups were 137.8 ± 65.8 mg/L, 141.4 ± 53.8 mg/L, 153.6 ± 49.3 mg/L, and 121.9 ± 55.9 mg/L, respectively. Two groups were determined based on the method of birth: cervical vaginal (vaginal birth) and the cesarean section. The measured OPN levels were 160.6 ± 48.8 mg/L and 99.9 ± 48.5 mg/L, respectively. For the BMI, mothers were categorized into pre-pregnancy and post-partum groups. This was examined under the headings of underweight, optimal, overweight, and obese in both groups. The pre-pregnancy results were measured as 140.6 ± 37.9 mg/L, 143.6 ± 53.6 mg/L, 111.9 ± 56.8 mg/L, and 159.1 ± 73.5 mg/L, respectively. The underweight was not measured in the post-partum group, and the remaining results were 156.4 ± 46.2 mg/L, 140.8 ± 61.2 mg/L, and 78.9 ± 28.8 mg/L, respectively. Three groups were determined based on weight gain during pregnancy: insufficient, adequate, and excessive. The corresponding amounts of OPN were 158.2 ± 40.3 mg/L, 149.0 ± 60.4 mg/L, and 119.8 ± 57.4 mg/L. Finally, OPN concentrations were examined according to smoking status. In this study, two groups were formed: pre-pregnancy and post-partum/lactation. In the pre-pregnancy group, mothers were categorized into groups according to their cigarette consumption before and during pregnancy. The OPN level of mothers who smoked before pregnancy was 102.0 ± 41.4 mg/L, while those who did not smoke had a level of 160.4 ± 53.8 mg/L. For mothers who smoked during pregnancy, the level was 103.1 ± 50.6 mg/L, while it was 143.8 ± 55.9 mg/L for mothers who did not. The amount of OPN for mothers who smoked in the post-partum/lactation group was 98.3 ± 49.2 mg/L, while for those who did not smoke, it was 144.1 ± 55.5 mg/L. When these results were examined, it was found that mothers who smoked had significantly lower OPN levels compared to non-smokers. The amount of OPN in breast milk may vary based on maternal factors. In addition, these statistical changes may impact the growth, development, and immunity of infants [70]. Another study was conducted in Japan. Cytokines in human milk were examined in 1989 and 2013. Colostrum samples were taken from 48 breastfeeding Japanese mothers, and mature milk samples were taken from 49 mothers. The amount of OPN in colostrum measured in 1989 was approximately 318.1 mg/L, while in 2013 it was 137 mg/L. At the same time, the amount of OPN in mature milk measured in 1989 was 300.8 mg/L, while in 2013, it was 280 mg/L. The amount of OPN present in colostrum in 1989 was found to be significantly higher, but no difference was observed in mature milk [86].

As a result of these studies, it has been found that the amount of OPN varies depending on maternal factors and geographical regions, and these changes can affect the growth, development, and immune system of infants. However, the reasons why there are different amounts of OPN in different geographical regions are not stated. More detailed research is needed on this issue. 

## 4. Biological Functions of Osteopontin

**Figure 2:**
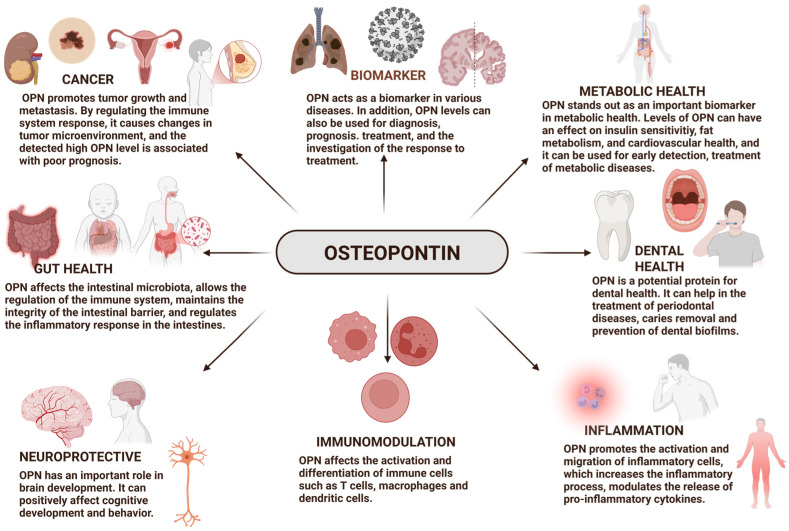
Functions of osteopontin [22,23,24,25,28,29,30,31,32,33,34,35,36,37,38,39,40,87,88,89,90,91,92,93,94,95,96].

OPN, a pleiotropic protein, plays significant roles in all vertebrates. OPN plays a role in many biological processes (Figure 2) [42]. It also has biological activities in many fields such as inflammation, biomineralization, biomarkers [35,37,38,39,40], dental health [28,29,30], cancer [87,88,89,90], diabetes, and wound healing, among others [42]. Thanks to the properties of OPN, it has been shown that it can play important roles in the diagnosis and treatment of many diseases (Table 1) [35,37,38,39,40,87,88,89,90]. It also allows for the development of various treatment strategies. In this section, OPN is discussed from multiple perspectives, and its potential in various areas is highlighted, including its use as a biomarker; its potential in cancer treatment; and its influence on gut health, microbiota, the immune system, dental health, brain development, and cognitive functions.

### 4.1. Osteopontin as a Biomarker

OPN is a secreted protein conserved across all vertebrates. Initially discovered in the extracellular matrix of bovine bone and linked to bone metabolism [5], it is now recognized for its broader roles in inflammation, immune regulation, cancer progression, and tissue repair. OPN influences cellular functions such as migration, adhesion, and differentiation by modulating survival and motility pathways [97,98]. In recent years, its expression levels in tissues and serum have been investigated for their diagnostic and prognostic value, highlighting its potential as a biomarker of various diseases [34,35,36,37,39,40,41]. OPN is a versatile protein involved in the differentiation of Th1 and Th17 cells and in tissue remodeling during inflammation. A study aimed to use OPN to determine the diagnostic and prognostic value of the disease in ILDs included a study involving 344 ILD patients, where approximately 70% were men, and 66.7% had a smoking history. Serum OPN levels were measured by ELISA, and the expression of OPN RNA was analyzed by qPCR. In addition, serum OPN levels in 140 healthy individuals were examined. As a result, it was found that the median value of the serum OPN levels of ILD patients was 1.05 ng/mL, while this value was 0.81 ng/mL in healthy individuals, and the OPN levels of patients were significantly higher. In addition to this condition, it was found that the OPN level increased significantly compared to other ILD patients (0.99 ng/mL) in cases where patients died or had a lung transplant (1.15 ng/mL). Additionally, it has been noticed that the survival time of patients worsened if the OPN level was higher than 1.03 ng/mL. No significant difference was found between serum OPN levels and RNA expression among different ILD diagnoses. Therefore, a high OPN level can be used as a biomarker to help detect ILD patients [34]. HCC is one of the most prevalent malignant tumors commonly found in Egypt. This disease, which has a poor prognosis and survival rate, is among those that are difficult to treat. For this reason, the early diagnosis of HCC disease was very important for patients. It was evaluated whether OPN, secreted by T cells, osteoblasts, and macrophages could be a potential marker in HCC patients with hepatitis C virus (HCV). A total of 140 patients, aged between 27 and 79, were included in this study. Four groups were formed: the first group consisted of 20 healthy controls; the second group included 40 patients with HCV; the third group had 40 patients with HCC; and the fourth group was comprised of 40 patients with both HCV and HCC. OPN expression was evaluated by Western blotting and ELISA methods. The OPN level was measured as 0.01189 ± 0.0075 mg/L for the first group, 0.04734 ± 0.00271 mg/L for the second group, 0.201 ± 0.00585 mg/L for the third group, and 0.25152 ± 0.01963 mg/L for the fourth group. As a result, it was shown that the OPN levels were significantly elevated in HCC patients. In other words, they could be distinguished from individuals without HCC. The use of OPN for HCC is very important for early diagnosis and survival monitoring. CA.CA19.9, CEA, and AFP are also biomarkers used in cancer diagnoses, but according to the ROC curve analysis, OPN was found to have a higher sensitivity and specificity than these biomarkers, and the AUC (Area Under the Curve) value was reported as 0.913. The OPN shows that it can be considered as a potential biomarker for the early diagnosis of HCC [36]. In one study, the potential of OPN to be used as a biomarker to predict undesirable conditions such as inflammation and bone deterioration that occur after MBS was investigated. The data from five different studies were used, and the OPN levels of the patients were evaluated before and after the surgery. As a result, it was found that OPN levels increased significantly after MBS. In other words, it has been predicted that OPN levels can be used as a marker for monitoring inflammation and bone health disorders in patients undergoing bariatric surgery. Additionally, the development of treatment methods targeting OPN has been shown to play an important role in the elimination of inflammation and bone deficiencies [41]. OPN has been found to play an important role in several biological processes, including atheromatosis and VC, as shown in numerous studies. Furthermore, research has revealed that OPN contributes to pro-inflammatory processes in ASCVD and intricately interacts with VC. From a clinical perspective, most studies support the idea that OPN serves as a biomarker for the presence, severity, and prognosis of CAD [37]. In a study conducted in 2024, researchers investigated the relationship between OPN and post-COVID-19 symptoms, lung function, and imaging abnormalities. Data from 122 hospitalized COVID-19 patients and 181 visitors were recorded at 4–84 weeks and circulating OPN levels were determined by ELISA. An increased level of circulating OPN in hospitalized patients was associated with persistent COVID-19 symptoms. Patients who showed symptoms had higher OPN levels compared to those who did not. It was also found that patients with dyspnea (shortness of breath) had higher OPN levels compared to those without. Moreover, OPN levels were higher in patients with more than one symptom, evaluated by the modified-Medical Research Council (m-MRC) scale to score dyspnea severity. Patients with a score greater than one had higher OPN levels than those with a score between 0 and 1. In summary, increased OPN levels in circulation were associated with severe dyspnea and a decline in quality of life [39]. Researchers observed the effects of OPN on mild cognitive impairment (MCI), Alzheimer’s disease (AD), or pre-symptomatic AD. The cerebrospinal fluid (CSF) OPN level was measured to investigate associations with AD and synaptic biomarkers. Two groups participated in the study: 167 cognitively unimpaired (CU) individuals and 399 individuals with MCI. These participants were selected from the PREVENT-AD project and the Alzheimer’s Disease Neuroimaging Initiation (ADNI) cohort. The levels of amyloid beta (Aβ) and tau proteins, along with changes in OPN levels, were examined in the participants. Survival analyses were performed to investigate the relationship between conversion rates to AD and OPN levels. As a result, CSF OPN levels showed a significant direct positive association with synaptic biomarkers. In addition, the PREVENT-AD and ADNI studies reported that individuals with a positive Aβ 42/40 ratio and elevated tau protein levels exhibited a significantly faster progression to AD. Moreover, high CSF OPN levels were associated with an accelerated conversion to AD in these individuals. Following the autopsy studies conducted on individuals who were found to have AD, it was shown that the level of OPN in the frontal cortex region of the brain increased significantly [40].

The basis for the classification of OPN as a biomarker lies in its biological functions, as well as measurable changes that occur under pathological conditions, and their relationship to disease onset and progression. Therefore, OPN is an important glycoprotein associated with many diseases, and serves as a biomarker, drawing attention due to its increased levels in various pathological conditions. Evidence from clinical trials indicates that OPN reflects disease progression and prognosis. This is especially true for long-term kidney diseases, ILDs, coronary artery diseases, post-COVID-19 symptoms, and more. Furthermore, the correlation of increased CSF OPN levels with synaptic biomarkers and conversion to symptomatic AD in the early detection of diseases of patients with HCC strengthens the evidence for the prognostic value of OPN. The role of OPN as a potential biomarker is important for diagnosing disease progression and evaluating patients’ responses to treatment. The study of OPN levels in the future will play a significant role in the advancing of new treatment strategies. Future research is expected to further develop and explore the role of OPN as a biomarker.

### 4.2. Osteopontin and Cancer

One of the leading causes of death in the world is cancer. According to GLOBOCAN 2020, produced by the International Agency for Research on Cancer, approximately 19.3 million new cases and 10 million deaths were recorded in 2020. It is expected that there will be 28.4 million cases due to a 47% increase [99]. Although research on tumors is progressing, it is still difficult to develop anticancer drugs. For this reason, a better understanding of the molecules that affect tumor progression is necessary for the development of new treatment strategies. This section examines the effect of OPN on cancer [42]. Numerous cells including tumor cells, macrophages, T cells, B cells, and dendritic cells produce the OPN protein. Several studies demonstrate the association of OPN with cancer; however, its functional contribution remains unproven [100]. Different receptors interact with OPN, initiating various cancer-related signaling pathways that support cancer cells’ growth, migration, invasion, and survival. OPN also alters the behavior of immune cells around tumors, which may weaken the immune system’s ability to eliminate tumors and lead to immunosuppression [101]. In a study conducted on 346 gastric tumor samples to examine the expression of OPN, E-cadherin, β-cadherin, and cyclooxygenase-2, immunohistochemistry was used to facilitate these analyses. Peritoneal relapses, dispersed histotype, and elevated histological grade were associated with the overexpression of OPN. According to the results, the high expression of OPN was closely correlated with metastasis, recurrence, and survival in patients who have undergone gastric cancer [87]. In another study, prostate tissue samples from 40 people with prostate cancer and 30 patients with benign prostate hyperplasia were examined. The expression of OPN splicing isoforms was analyzed in these prostate tissue samples. In this analysis, anti-OPN antibodies were evaluated by an immunohistochemical staining test, and based on this study, it is suggested that OPN may be useful in diagnosing and monitoring prostate cancer due to its significantly high expression in this type of cancer [102]. The study involved 33 patients with EEC and 30 patients with OEC. The OPN levels of EEC and OEC cancer patients were analyzed and compared by quantitative reverse transcriptase-polymerase chain reaction (RT-PCR) and quantitative PCR. As a result, it was found that the release of OPN was lower in early-stage cancer patients than in advanced-stage cancer patients; however, this difference was not statistically significant [88]. OPN is a protein associated with hypoxia, and in a study, plasma samples were taken from 172 patients to examine the relationship between the amount of OPN in their blood plasma (which is associated with hypoxia) and their health status. As a result of the study, regardless of the treatment method, the survival rates of patients with low OPN levels were significantly better than those with high levels. This suggests that patients with low OPN levels have a higher chance of survival [90]. A recent study examined some aspects of the transcriptomic expression profile of SPP1, namely OPN. These included conditions under which it was activated or dysregulated, related biological pathways, and different mechanisms that altered its function. Tissues from breast, prostate, kidney, and skin cancer were studied to observe how differences in splicing patterns affect protein expression and function. As a result, OPN was found to be expressed at significantly higher-than-normal levels in most of the tissues. In addition, it was observed that OPN expression was particularly elevated at the advanced tumor stage in kidney and skin cancers. The application of treatment methods targeting genes associated with OPN led to a significant decrease in its effect on these cancer types. The fact that these treatments can improve patients’ chances of survival and their response to therapy by activating the immune system is one of the most important significant findings [89].

Nowadays, cancer still causes morbidity and mortality. For this reason, the availability of specific biomarkers is vital for the development of early detection and treatment methods. In this context, the OPN protein, involved in the pathogenesis and progression of cancer may play a very important role [42]. Increased OPN expression in various types of cancer has a significant role in cancer progression. Furthermore, researchers have observed a strong relationship between the level of OPN and patient survival. Considering all this information, OPN has the potential to serve as a biomarker. Thanks to future studies, early diagnosis and treatment methods using OPN can be improved, allowing for a better understanding of the relationship between OPN and cancer [89].

### 4.3. Osteopontin and Gut Health

OPN functions as a potent cytokine with a significant influence on intestinal development. Studies have demonstrated that its expression in epithelial cells contributes to the regulation of intestinal barrier integrity in both healthy and diseased conditions. The presence of OPN in the healthy gut further supports its role in maintaining immune homeostasis within the intestinal environment [103]. The level of inflammation correlates with the low OPN expression. The response of mucosal T cells to OPN differs between healthy and diseased individuals. As seen in Crohn’s disease, OPN enhances the pro-inflammatory cytokine response due to insufficient IL-10 production. However, an increase in the IL-10 level is effective in maintaining homeostasis in healthy individuals [104]. In an animal study, the observed differences in developmental processes between formula-fed and breast-fed newborns were thought to be due to the reduced milk OPN content in the formula. Premature piglets were used as the model, and on the eighth day, the OPN group had significantly higher monocyte and lymphocyte counts as well as higher villus-crypt ratios than the raw milk group. However, the intestinal functions were similar between the two groups [92]. In another animal study, the impacts of milk OPN on improving intestinal function in pregnant rats fed an HFD were examined. Pregnant rats were given bmOPN supplementation at a dose of 6 mg/kg body weight. Thanks to this reinforcement, bmOPN supplementation significantly reduced colon inflammation in female rats fed with HFD. Adding bmOPN notably increased the expression of proteins such as ZO-1 (zona occludens-1) and claudin-4, which maintain intestinal integrity. These proteins are significant molecules that contribute to the structure and function of the intestinal barrier. BmOPN also caused significant changes in the intestinal microbiome of rats; specifically, it increased the relative abundance of Bacteroidetes, while decreasing the abundance of bacterial taxa such as Proteobacteria, Helicobacteraceae, and Desulfovibrionaceae. Short-chain fatty acid levels were significantly higher in rats that were fed an HFD and also received bmOPN supplementation compared to those that did not receive the bmOPN supplement. These fatty acids play an essential role in intestinal health and reducing inflammation. Adding bmOPN may have altered four important biochemical processes in the microbiome of pregnant rats fed an HFD. One of these processes was the production of bile acid. Moreover, bmOPN supplementation significantly increased the levels of several liver-derived bile acids, including taurochenodeoxycholic acid, tauroursodeoxycholic acid, and taurohyodeoxycholic acid [93]. In an animal-based study, the goal was to evaluate the bioactivity of bmOPN using knockout mouse models. The effect of bmOPN on intestinal growth was observed by nursing wild-type pups with WT, KO, or DKO dams supplemented with bmOPN for 1–21 days. As a result, it was found that orally administered bmOPN was partially sensitive to gastrointestinal digestion in vivo. Post-natal day 10 revealed similar notable effects on mouse milk OPN (mmOPN), which promoted small intestine growth, as shown by the histological analysis of duodenal villus height and crypt depth. In line with these findings, it was determined that adding bmOPN to infant formulas may be beneficial [35]. In a study conducted on piglets, researchers exposed 96 piglets to four distinct dietary treatments. These treatments were as follows: standard milk (CTR) for the first group; probiotics and prebiotics (galacto-oligosaccharides 4.36 g/L and human milk oligosaccharides 0.54 g/L) together with CTR for the second group; OPN (0.43 g/L) with CTR for the third group; and finally, prebiotics and OPN with CTR for the fourth group, which was the control group. The researchers recorded the piglets’ data over a period of 15 days. The results demonstrated that adding synbiotic OPN to piglet milk formulas, or OPN alone, may alter the formation of the intestinal microbiota, the development of digestion, and most importantly, the attachment response of the newborns. Thanks to these supplements, diarrhea attacks decreased in piglets, beneficial microbiota types were slightly promoted, and dysbiotic species declined. Most importantly, OPN supplements increased the concentration of SCFAs, decreased ammonia levels, and reduced the intraepithelial lymphocyte counts; however, these results were not statistically significant [94]. In an animal-based study on OPN’s effectiveness in digestive system inflammation, OPN-null mice with DSS-induced acute colitis resulted in greater tissue damage and lower TNF-α production, which was associated with an increase in non-apoptotic cell death in the colonic tissue. Although they had significantly larger spleens, a decrease in immune cells was observed. The pro-inflammatory cytokine response indicated that the number of neutrophils decreased significantly, while increased tissue myeloperoxidase levels contributed to enhanced inflammation. As a result, OPN-null mice showed more severe signs of colitis, revealing that OPN is necessary for mucosal protection [105].

In animal studies, OPN supplementation has been shown to reduce inflammation and support the microbiota, while deficiencies have been associated with intestinal damage and dysbiosis. In addition, thanks to OPN supplementation, the expression of tight junction proteins (ZO-1, Claudin-4) increased, and the synthesis of SCFA, leading to a reduction in inflammation. These findings indicate that OPN has great potential in maintaining intestinal integrity and microbiota. However, more research is needed, particularly in human models, to fully understand the effects of OPN on gut health and the treatment of intestinal diseases.

### 4.4. Osteopontin and Immunological Effects

OPN is referred to as an ETA-1, which is expressed when T cells are activated [106]. OPN is associated with numerous immune processes, including inflammatory conditions such as cancer, allergic reactions, tissue damage, and autoimmune diseases like multiple sclerosis (MS) and rheumatoid arthritis [63]. It is expressed in various body fluids, tissues, and cells, including macrophages and dendritic cells [22,63].

**Figure 3:**
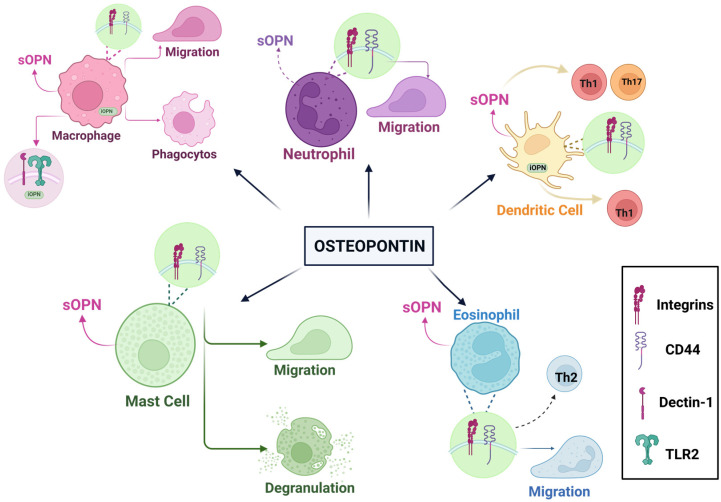
In different cell types, OPN expression and its effects. OPN receptors, integrins, and CD44 are commonly synthesized on the surfaces of the cell types shown here and are analyzed in detail in the peripheral immune system. sOPN supports cell migration and effector functions such as cell proliferation, immune response, and inflammatory processes. Also, iOPN is a form of OPN found inside the cell and is involved in regulating immune responses and signal transmission. In macrophages, sOPN supports phogocytosis and cell migration; in dendritic cells, sOPN is involved in the migration of Th1, Th2, and Th17 cells. SOPN promotes migration degranulation in eosinophil, neutrophil, and mast cells [63].

SOPN has many important functions in macrophages, such as cell migration, proliferation, phagocytosis, and MMP production. Thanks to sOPN, monocytes transform into macrophages via integrins. IOPN strengthens and regulates immune responses in macrophages. It regulates antiviral responses in peritoneal macrophages. Dendritic cells produce sOPN, which enhances TH1 polarization by aiding in the production of TNF-α and IL-12. In addition, iOPN promotes IFN-I production while regulating Th17 responses in dendritic cells. It also facilitates interactions between MSCs and dendritic cells, acting as a repressor of the OPN protein in the presence of pro-inflammatory cytokines. Neutrophils, the most abundant leukocytes in the bloodstream, also produce OPN, albeit in smaller amounts than macrophages. Independent of CD44, sOPN increases the migration of neutrophils. However, in fulfilling this role, it can occasionally cause harm to the host cells. Eosinophils also produce and secrete OPN, contributing to airway angiogenesis in asthma. One study, using sputum samples from asthmatic patients, revealed a correlation between OPN levels and the eosinophils counts, concluding that sOPN could serve as a biomarker for disease detection [63,107], as shown in the Figure 3. Another study analyzed the amounts of OPN in breast milk, bovine milk, and infant formula. These measurements revealed an average of 138 mg/L of OPN in breast milk. This measurement led to the conclusion that it represented 2.1% of the overall protein in breast milk (weight/weight). At the same time, the amounts of bmOPN and infant formula were approximately 18 mg/L and 9 mg/L, respectively. It has been found that OPN can increase the production of IL-2 and thus affect the immune response. Additionally, researchers analyzed the amounts of OPN in 3-month-old infants using plasma samples from both pregnant and non-pregnant women. In this study, it was found that the levels of OPN in plasma taken from 3-month-old babies and umbilical cords were 7–10 times higher, which is considered significant. It was considered that high OPN levels are very important for infants and can increase cytokine production in newborns [24]. In another study, IL-10-knockout mice were used to investigate the role of OPN in the development of intestinal inflammation. When OPN/IL-10 and IL-10 were then compared, researchers observed a faster development of colitis in OPN/IL-10 mice. This indicates that insufficient OPN disrupted gut microbiota and the normal functions of macrophages, thereby worsening inflammation in the gut. These findings were statistically significant, highlighting the crucial role of OPN in maintaining intestinal immune homeostasis [22]. Another study examined the relationship between B cell aggregates in the central nervous system (CNS) and OPN. B cells in MS brain tissue expressed OPN. One of the most striking findings was the different effects of OPN on B cells. One of these effects was that OPN down-regulated the expression of the CD80 and CD86 helper stimulating molecules and had the ability to reduce B cell activation. Another effect was that it could help B cells form clusters, which may enhance B cells’ activation. On the other hand, OPN had both anti-inflammatory and pro-inflammatory effects on autoimmune disease. It could stimulate the production of IL-6 and autoantibodies by affecting B cells; this was due to the pro-inflammatory cytokine properties of OPN. Among its anti-inflammatory properties was the potential to regulate tissue repair. In addition, OPN is associated not only with autoimmune diseases, but also with chronic neuroinflammation and has been associated with the clustering of B cells. These anti-inflammatory and pro-inflammatory properties of OPN may provide potential for future studies. More importantly, the study’s findings were statistically significant, potentially enabling the identification of new treatment methods for patients with chronic neuroinflammatory diseases [23]. 

A clinical study investigated the immunological activity of OPN. Three groups were used in the research: standard formulas, 65 mg OPN/L (about 50% of the hmOPN concentration), and 130 mg OPN/L (equal to 100% of the hmOPN concentration). Milk containing these amounts was given to 1-, 4-, and 6-month-old babies who were fed spontaneously, and the obtained data were compared. As a result of this comparison, it was found that formula-fed infants had higher levels of the pro-inflammatory cytokine TNF-α in their plasma than breast-fed infants. It was also found that TNF-α levels were lower in infants fed with formula containing added OPN. More importantly, the way babies fed with OPN-enriched milk used amino acids and their cytokine responses were similar to those of breast-fed babies. One of the most important things to note is that babies who took OPN supplements had fewer feverish days (pyrexia) than babies who did not take any supplements. Additionally, babies fed milk containing 130 mg OPN/L had higher T cells compared to babies fed other food groups. These findings have shown that OPN also plays a significant role in immune development [25]. In another study, premature pigs were given OPN at a body weight of 46 mg/kg per day, while some pigs were given raw bovine milk, and the relationship between the groups was examined. Although the pigs exhibited similar immune statuses, significantly higher levels of monocytes and lymphocytes were found in the intestines of OPN-fed pigs after the eighth day compared to those fed raw bovine milk [92].

As a result, based on the studies and findings, it was concluded that giving OPN to babies benefits their immune systems and may be important for discovering new ways to treat certain diseases.

### 4.5. Dental Health

During the formation process of a tooth, OPN is located in non-mineralized tissues and functions to reduce or stop mineralization during the production of periodontal ligaments [108]. OPN, which is involved in the healthy development and repair of mineralized tissues, also helps maintain tissue integrity by ensuring cell–matrix and matrix–matrix/mineral adhesion [109,110]. OPN is a highly phosphorylated sialoprotein that serves a noteworthy function in the mineralized extracellular matrices of teeth. Additionally, OPN has been shown to play an important role in the fluoride remineralization process. One study investigated how OPN influenced the process of enamel demineralization. Enamel lesions with a depth of 180–200 µm were generated and exposed to *Streptococcus mutans* for 21 days in a calcium phosphate solution containing 0.499 mg/L OPN. The results showed that in the presence of OPN, demineralization was counteracted, as mineral loss and repair occurred simultaneously. In contrast, fluoride alone acted only on the surface. OPN also appeared to stabilize fluoride ions, suggesting it may significantly enhance fluoride’s effectiveness in preventing deeper demineralization [29]. In another study, the aim was to examine the relationship between hydroxyapatite surfaces, and to evaluate OPN’s potential to prevent bacterial adhesion to the tooth surface. The preventive effect of OPN on plaque formation was investigated in vitro using hydroxyapatite discs, which served as a model for tooth enamel. These discs were immersed in an OPN solution and subsequently incubated with saliva. The number of bacteria forming plaque on the surfaces was then analyzed. The result showed that although the thickness of the OPN film was reduced after exposure to physical and chemical treatments such as rinsing and sodium dodecyl sulfate application, OPN remained bound to the surface and formed a stable coating. This indicates that OPN may help bacterial adhesion by creating a long-lasting protective layer on the tooth surface, suggesting its potential role as a beneficial biomolecule for maintaining oral health, and that the findings of the study were significant [30]. In a different study, the binding properties of bacteria to saliva-coated OPN surfaces were examined. *Actinomyces naeslundii*, *Lactobacillus paracasei* subsp. *paracasei*, and *Streptococcus mitis* bacteria were used in the study. It was found that OPN was the most effective protein inhibiting adhesion, and this finding was significant [28]. 

In summary, OPN holds promise for dental health due to its ability to promote remineralization and inhibit bacterial colonization. It has potential applications in the treatment of periodontal diseases, caries prevention, and the control of dental biofilms. Formulations containing OPN, such as toothpaste and mouthwashes, have demonstrated clinical benefits by enhancing enamel repair and reducing microbial adhesion. Future research aims to incorporate OPN into dental repair materials or implant coatings to improve oral tissue integration and overall therapeutic outcomes. Its antimicrobial and regenerative properties may contribute to long-term oral health and a reduced infection risk.

### 4.6. Osteopontin and Brain Development: Cognitive Function

OPN, a pro-inflammatory cytokine, is expressed in various cells and tissues, including activated macrophages and T–lymphocytes. It is a key component of the extracellular matrix in the CNS. Notably, OPN levels increase in response to inflammation and injury within the CNS [111]. Research has demonstrated that OPN is involved in several neurological conditions, including MS [112,113], Parkinson’s disease [114], AD [40,115], and other neurodegenerative disorders, while also promoting the progression of these diseases [116]. In one study, researchers investigated the effects of bmOPN on cognitive development and brain health. In total, 20 male pigs were fed two different diets for up to 34 days after birth using a standard soy protein isolate-based milk powder substitute and an OPN-supplemented diet. Brain development was analyzed by an MRI, while learning and memory characteristics were assessed by a novel object recognition task. Pigs on the OPN-supplemented diet reacted to the object they saw more quickly, and relative brain volumes increased in specific brain regions of the pigs in this group. In addition, the study concluded that regular consumption of OPN is safe in pig models, and these findings are statistically significant [31]. In another study, OPN, known for its protective properties for nerve cells and brain tissue in ischemic brain injuries, was found to be neuroprotective under HI conditions. Researchers investigated whether OPN could correct neurological damage by blocking the right carotid artery of the rat pups to prevent blood flow to one side of the brain. The brain injury was induced by placing the pups in a low-oxygen environment to study the effects of OPN supplementation on HI. The results showed that OPN levels decreased with age, but endogenous OPN was produced again as a self-protective response to brain damage, peaking at 48 h. Additionally, exogenous OPN was found to reduce the volume of damaged brain tissue and alleviate neurological consequences, demonstrating significant effects. More importantly, OPN acted as an apoptosis inhibitor by suppressing caspase 3, improving neurological functions in long-term treatments [32]. In another study, the effect of OPN on SCI was investigated. It was observed that OPN levels significantly increased during the formation of SCI in SCI-induced mice. Researchers examined the levels of OPN and the distribution of cells in the spinal cord, along with the impact of OPN on neuropathic pain and motor ability. The study found that OPN exhibited the highest-level increase after SCI. Suppressing OPN levels worsened motor function and increased pain, demonstrating that OPN contributes to the healing process, supports the recovery of motor functions, and can suppress pain [33]. In another study, the effects of OPN on ischemic stroke were investigated. The research demonstrated that OPN reduces cerebral damage and supports recovery [117]. In another study, the effect of consuming diets supplemented with bmOPN on brain development was investigated. The results indicated that OPN supplementation had no effect on cognitive development [92]. Another study investigated the effects of milk-derived OPN on brain development using two groups of mice. One group was fed milk containing OPN, while the other group received milk without OPN. The analyses revealed that OPN from milk was transported to the brain, with a direct increase in OPN levels observed in the brains of mice consuming OPN-supplemented milk. Additionally, cognitive development was found to be worse in mice that were fed milk without OPN supplementation, indicating that the differences observed are statistically significant [118]. Considering all the studies, it is evident that OPN used as a supplement positively affects cognitive development and behavior while supporting memory development. OPN can support the formation of new cells and serve therapeutic purposes in cases of brain damage.

## 5. Nutritional Potential of Osteopontin

Newborn babies need breast milk to protect against infections in the early stages of their lives. Milk is rich in nutrients and bioactive components [24,119,120,121,122,123,124,125,126,127]. It is very important for babies to be fed with breast milk in the first 6 months after birth, when the immune system has not yet developed [125,128]. Currently, it has been found that infants fed with formula contract more infections than infants fed with breast milk [129]. This is due to the lack of certain proteins with antibacterial, immunostimulating, and protective properties contained in the food consumed by formula-fed babies. Breast milk is produced to meet all the needs of a baby due to its structure and content; however, infants who cannot be fed with breast milk are fed with formula. To better mimic the composition of breast milk and support infant development, many studies have focused on enhancing the nutritional content of formula. One key component with potential immunological benefits is OPN, a multifunctional glycoprotein involved in diverse physiological processes. Notably, its concentration in human milk is significantly higher than in most other tissues and body fluids [24].

The concentrations of bmOPN, breast milk, and infant formula were analyzed. As a result of these analyses, OPN concentrations of approximately 138 mg/L in human milk, 18 mg/L in bovine milk, and 9 mg/L in infant formula were determined. This indicates that the amount of OPN consumed by formula-fed babies is quite low [24]. It has been shown that significant differences in gene expression occur in the intestines of breast-fed monkey babies. At the same time, it was noticed that half of the gene expression in babies who consumed formulas with bmOPN was similar to that of monkey babies who were fed breast milk. That is, OPN consumption has been shown to positively affect the intestines of infants. In addition, the regulation of cell migration by OPN and its interactions with integrins also support these results [95]. High plasma levels in newborn babies indicate that OPN is of critical importance for the infant. As in the case of a baby monkey, OPN consumption can support neonatal intestinal immunity and stimulate cytokine production. OPN also affects the high monocyte and lymphocyte levels in pigs consuming OPN, as well as the deeper villi-crypt level. This has a positive effect on the immune system and intestinal health resulting from OPN consumption [92]. Also, the use of OPN supplements by women during pregnancy limited the occurrence of colon inflammation and supported the integrity of the intestinal barrier. In addition, its presence in the intestinal flora supports the microbiota population by inducing the production of SCFA and promotes the synthesis of bile acids, thereby improving intestinal health [93]. Some of the milk proteins, such as OPN, are resistant to digestive enzymes, and mobile proteins that are biologically active have been identified [130]. The protective effect of OPN is based on its ability to regulate and maintain the structural and functional integrity of the stomach, intestines, as well as reducing liver inflammation [131]. Additionally, in another study, Lacprodan^®^ OPN-10 was found to be non-mutagenic in bacterial reverse mutation testing, non-clastogenic in chromosomal aberration tests, and showed nogenotoxicity according to in vivo micronucleus testing. In addition, according to a 13-week subchronic toxicity study, the highest safe dose was determined as 1208 mg/kg body weight per day for male mice and 1272 mg/kg body weight per day for females. Also, the application of 2.50 g/kg Lacprodan^®^ OPN-10 applied to pregnant females did not negatively affect the pregnancy rate and fetal development. As a result, the amount of Lacprodan^®^ OPN-10 found suitable for breastfed babies in this study was an average of 25 mg/kg body weight. More importantly, it was stated that even 35 times more than the recommended amount is not harmful, demonstrating that OPN consumption is safe for infants [132].

**Figure 4:**
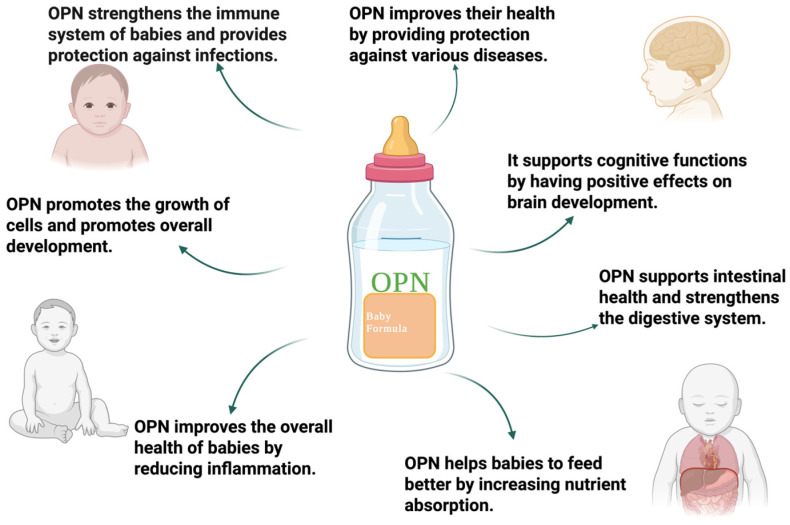
Nutritional potential of osteopontin.

Given these results, it has been determined that OPN plays important roles in supporting human health, especially in regulating the growth development and immune responses in infants. In particular, feeding babies who cannot be breastfed with OPN-supplemented foods is very critical for their development, as mentioned in the Figure 4. As a result, OPN is a potential protein that can be used in infant formula; however, more studies are needed before it can be implemented.

## 6. Conclusions and Future Remarks

OPN is a significant phosphoglycoprotein involved in various biological processes, including immune regulation, tissue regeneration, infection response, and fetal development. Its partial resistance to intestinal digestion allows it to enter circulation and support systemic physiological functions. Research highlights its potential as a biomarker, particularly for the early diagnosis and treatment of inflammation-related conditions such as cancer. Variations in OPN levels in human milk across geographical regions and maternal factors underscore the need for further studies to clarify these influences. Supplementation studies show that OPN supports intestinal development, enhances gut microbiota composition, and may influence cognitive functions in infants. These findings suggest that an OPN-fortified formula could be beneficial, especially for non-breastfed infants. Moreover, its roles in regulating intestinal inflammation and neurological function position OPN as a promising candidate for future therapeutic applications.

In summary, OPN is a biologically active molecule with expanding therapeutic potential. Ongoing research into its mechanisms and applications may pave the way for novel strategies in disease diagnosis, prevention, and treatment.

## Figures and Tables

**Table 1:** Reported Effects of Osteopontin in In vitro and In vivo Studies.

Function	Dose	Effect	Study Design	Ref
Neuroprotective	The OPN-supplemented formula was designed to use 250 mg/L OPN.	BmOPN supplementation enhanced the relative volume of various brain regions and changed behaviors in the novel object recognition task.	In vivo	[31]
Neuroprotective	OPN (0.03 μg or 0.1 μg) was administered intracerebroventricularly at 1 h post-HI.	Exogenous OPN reduced infarct volume and augmented neurological outcomes 7 weeks after hypoxic-ischemic (HI) injury, while an integrin antagonist inhibited OPN-induced neuroprotective.	In vivo	[32]
Neuroprotective	-	The possible advantages of an OPN increase in improving locomotor function and alleviating neuropathic pain subsequent to a spinal cord injury (SCI).	In vivo	[33]
Gut Health	-	OPN deficiency worsened alcohol-related disease (ALD). Increasing OPN in intestinal epithelial cells may help maintain the intestinal microbiome, protect barrier function, and combat ALD.	In vivo	[91]
Gut, Immunity, and Brain Development	Sixteen premature pigs were fed diets with 46 mg/(kg·g) of OPN added.	Pigs fed OPN supplementation showed increased villus-crypt depth, higher monocytes and lymphocytes, similar cognitive development, and improved intestinal structure and immunity, according to T-maze test results.	In vivo	[92]
Gut Health	BmOPN was enhanced at a dose of 6 mg/kg body weight.	OPN supplements in pregnant women prevented colon inflammation and enhanced intestinal barrier function. They regulated intestinal population through short-chain fatty acid (SCFA) production and supported bile acid secretion.	In vivo	[93]
Gut Health and Immune Function	In total, 0.436 g/L OPN was added to the milk formulation.	OPN improved digestion, microbiota, and the immune system in piglets. Synbiotic tablets enhanced benefical bacteria and reduced harmful genera, lowering diarrhea. SCFA increased and reduced ammonia, preventing pathogen growth.	In vivo	[94]
Gut Health	Formula fed supplemented with 25 mg/L bovine OPN.	Newborn rhesus monkeys fed breast milk or OPN-supplemented bovine milk showed similar growth and gene expression, suggesting OPN may affect intestinal development.	In vivo	[95]
Gut Health and Brain Development	In total, 12 µg/g OPN was used as a supplement every morning.	BmOPN enhanced small intestine growth, inhibited TNF-α secretion, increased brain myelination, and supported cognitive development.	In vivo	[35]
Biomarker	-	OPN was associated with atherosclerotic cardiovascular diseases (ASCVD) and vascular calcifications (VC), had varying effects on diseases, and may be a biomarker for coronary artery disease (CAD).	In vitro and in vivo	[37]
Biomarker	-	OPN levels increased post-MBS, suggesting they may indicate inflammation and bone health in bariatric patients, and suggesting OPN-targeting treatments could address these issues.	In vivo	[41]
Biomarker	-	The study highlights the significance of examining OPN levels in COPD and pneumonia, suggesting that OPN may have potential biomarker properties.	In vivo	[38]
Biomarker	-	COVID-19 patients with increased OPN levels in their circulation and severe symptoms have been suggested as potential biomarkers.	In vitro	[39]
Biomarker	-	Elevated CSF OPN predicted faster AD progression and was increased in a frontal cortex of AD brains.	In vivo	[40]
Biomarker	-	Interstitial lung disorders (ILDs) patients had higher OPN levels than healthy individuals. Elevated OPN was linked to lower vital capacity and higher mortality or lung transplant rates, indicating the potential for identifying ILD patients.	In vivo	[34]
Biomarker	-	In Egypt, OPN was studied as a biomarker for diagnosing hepatocellular carcinoma (HCC) in HCV patients. It proved more effective and sensitive than other markers, indicating its potential for early diagnosis.	In vivo	[36]
Dental Health	-	OPN was effective as process-directing agents utilized in the polymerinduced liquid-precursor (PILP) process to stabilize and convey mineral ions, and the regulated remineralization had the potential to boost the performance of fluoride.	In vitro	[29]
Dental Health	Previous studies used 46 µM OPN, but in this study, 50 µM was used due to the unobserved optimal effect.	Research indicates that milk proteins, particularly OPN, effectively inhibited bacterial adhesion to saliva-coated surfaces, potentially enhancing oral health and preventing dental biofilm.	In vitro	[28]
Dental Health	The study determined the optimal OPN dosage for use, ranging from 100 mg/kg to 1000 mg/kg, with the most suitable amount being approximately 350 mg/kg.	It has been found that OPN could bind to hydroxyapatite surfaces, as well as prevent bacteria from adhering to the tooth surface.	In vitro	[30]
Immunomodulation	-	Colitis progressed faster in OPN/Interleukin-10 (IL-10) double knockout (DKO) mice than in IL-10 knockout (KO) mice, indicating that OPN deficiency worsens colitis. OPN expression was higher in IL-10 KO epithelial cells. In OPN/IL-10 DKO mice, *Clostridium* subset XIVa decreased, while cluster XVIII increased.	In vivo	[22]
Immunomodulation	In total, 2 µg/well (1 µg/mL) recombinant OPN was added to B cells.	OPN reduced B cell clusters in MS brain tissue, increasing neuroinflammation and IL-6 and autoantibodies. Human recombinant OPN down-regulated IL-6 and up-regulated IL-10 in B cells.	In vitro	[23]
Immunomodulation	-	A 3-month-old baby’s umbilical cord OPN plasma was 7–10 times higher than normal, indicating low OPN concentration in infants’ intestinal immune systems, limiting cytokine expression.	In vitro	[24]
Immunomodulation	Milk formulas with 65 mg OPN/L or 130 mg OPN/L added were used.	Infants fed 130 mg of OPN/L showed increased T cell and monocyte levels, potentially promoting immunity development and progression.	In vivo	[25]
Cancer	-	High OPN levels can lead to cancer, promote tumor growth, and spread, with gastric cancer patients’ survival predicted by OPN levels.	In vitro	[87]
Cancer	-	In particular, the OPN levels of EEC (endometrioid endometrial cancer) and OEC (ovarian endometrioid cancer) cancer types were similar to each other. It was found that the OPN level was higher in advanced tumors.	In vitro	[88]
Cancer	-	OPN expression was high in breast, prostate, kidney, and skin cancers, with even higher levels in advanced kidney and skin cancers. Treatment targeting these cancers resulted in decreased OPN levels.	In vitro	[89]
Cancer	-	OPN expression in non-small cell lung cancer (NSCLC) patients was linked to survival, disease development, and treatment responses, with strong OPN expression resulting in shorter lifespans.	In vivo	[90]
Metabolic Health	-	OPN played a crucial role in high-fat-diet-induced lipid deposition, regulating intestinal lipid and fatty acid metabolism, and potentially preventing dyslipidemia by altering gut flora.	In vitro and in vivo	[96]

## Data Availability

Not applicable.
